# Supplementary material for: Liquid-Based Diagnostic Panels for Prostate Cancer: The Synergistic Role of Soluble PD-L1, PD-1, and mRNA Biomarkers
Source: Int J Mol Sci. 2025 Jan 15;26(2):704. doi: 10.3390/ijms26020704 (PMC11765789; doi:10.3390/ijms26020704)
Supplement: Supplementary file 1 [file ijms-26-00704-s001.zip › ijms-3389919-supplementary.pdf]

Liquid-Based Diagnostic Panels for Prostate Cancer: The Synergistic Role of Soluble PD-L1, PD-1, and mRNA Biomarkers

Margarita Žvirblė[1,2], Ieva Vaicekauskaitė[1,2], Žilvinas Survila[2], Paulius Bosas[1], Neringa Dobrovolskiene[1], Agata Mlynska[1,3], Rasa Sabaliauskaitė[1,2], Vita Pašukonienė[1]

Supplementary materials

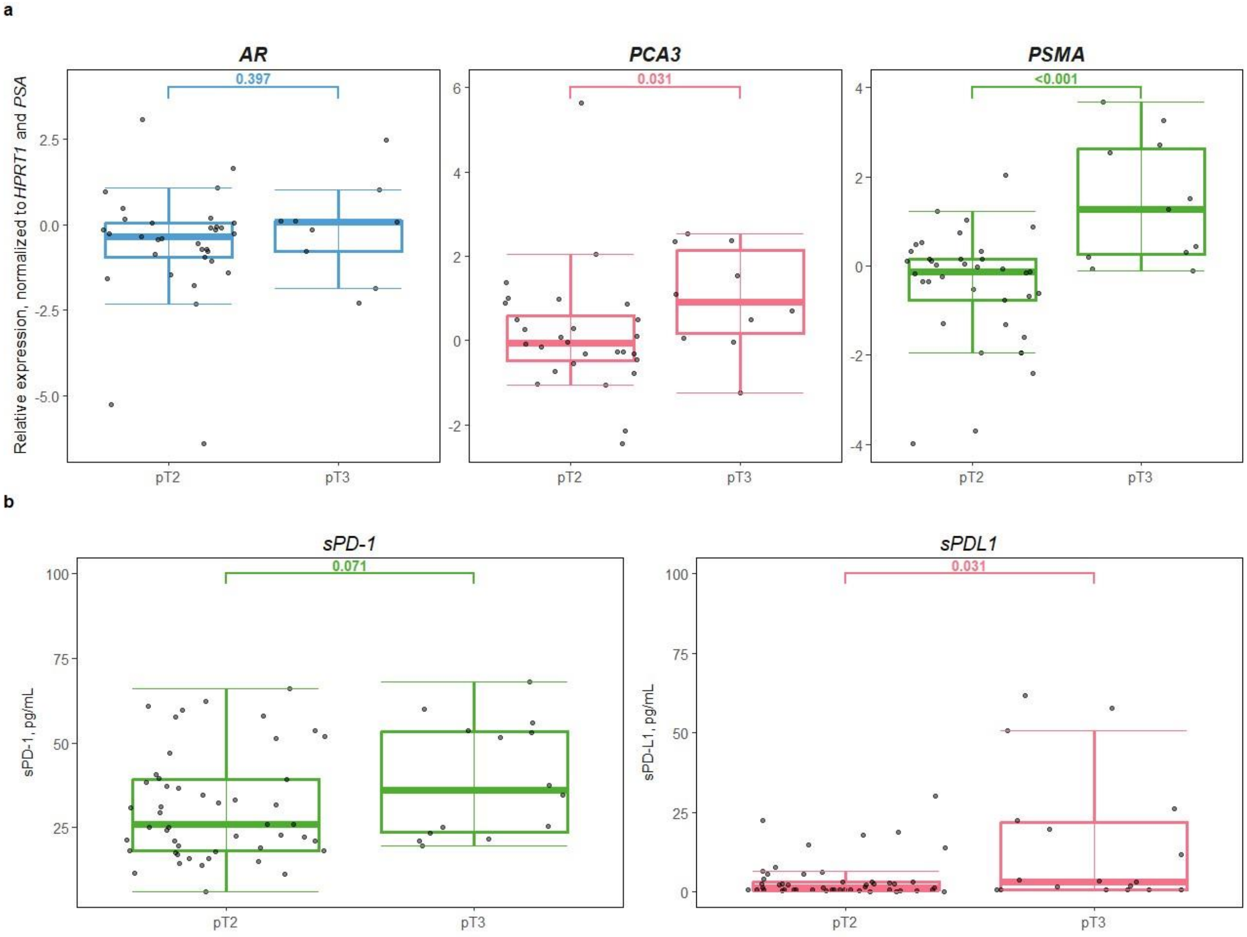

Figure S1 Relative expression values of *AR*, *PCA3*, and *PSMA* mRNAs as well as sPD-L1 and sPD-1 in clinically significant and insignificant PCa cases, according to pathological stage.

A Relative expression values of *AR*, *PCA3*, and *PSMA* mRNAs in association with tumor stage. *PCA3* $p < 0.05$ and *PSMA* $p < 0.001$. **B** sPD-L1 and sPD-1 levels in association with tumor stage. sPDL1 $p = 0.031$.

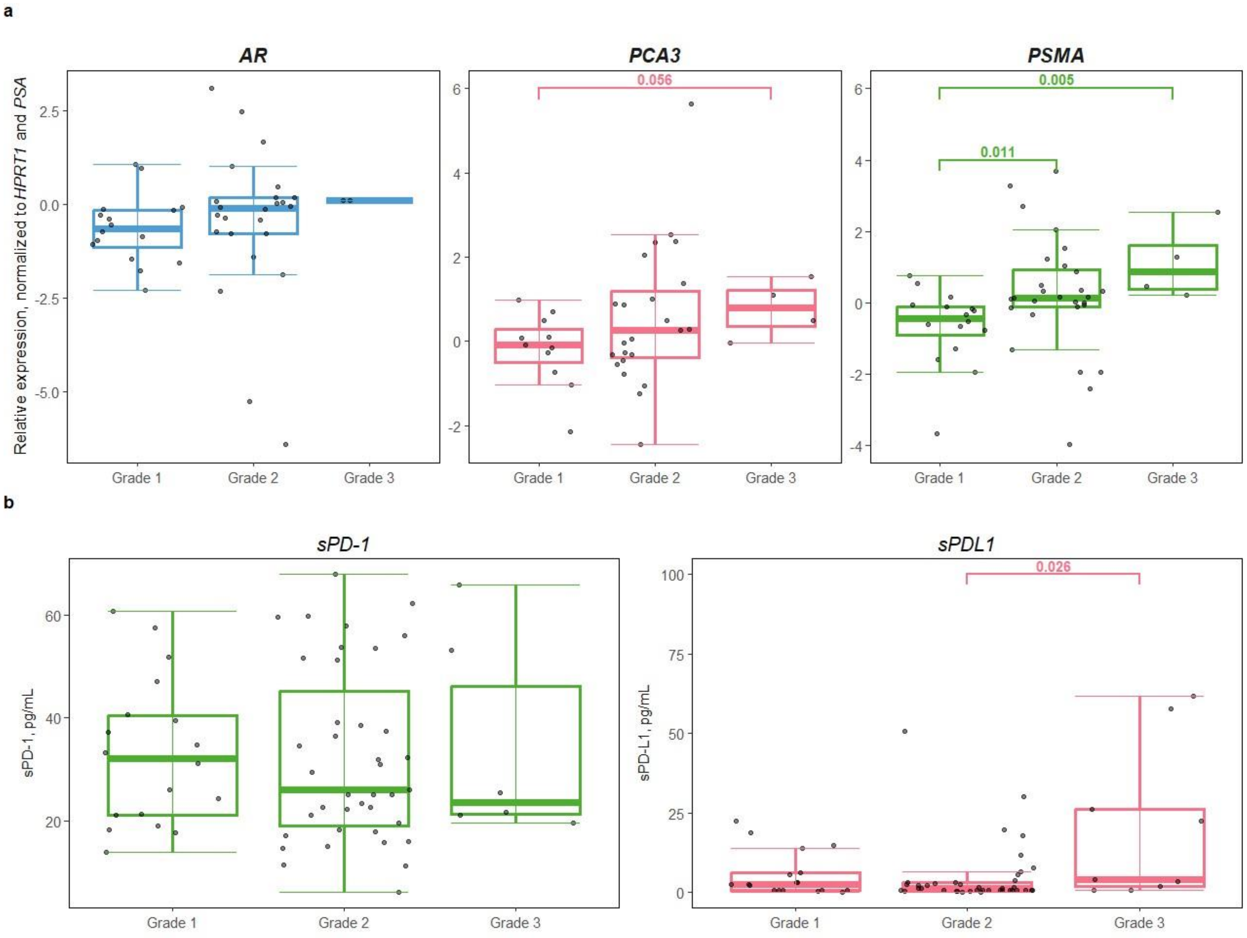

Figure S2 Relative expression values of *AR*, *PCA3*, and *PSMA* mRNAs as well as sPD-L1 and sPD-1 in association with ISUP grading.

A Relative expression values of *AR*, *PCA3*, and *PSMA* mRNAs in association with ISUP grading. Grade 1 *vs* grade 3 *PSMA* p = 0.005, grade 1 *vs* grade 2 *PSMA* p = 0.011. **B** sPD-L1 and sPD-1 levels in association with ISUP grading. Grade 2 *vs* grade 3 sPDL1 = 0.026.
